# Supplementary material for: Type 1 Corticotropin-Releasing Factor Receptor Differentially Modulates Neurotransmitter Levels in the Nucleus Accumbens of Juvenile versus Adult Rats
Source: Int J Mol Sci. 2022 Sep 16;23(18):10800. doi: 10.3390/ijms231810800 (PMC9505341; doi:10.3390/ijms231810800)
Supplement: Supplementary file 1 [file ijms-23-10800-s001.zip › ijms-1820773-supplementary.pdf]

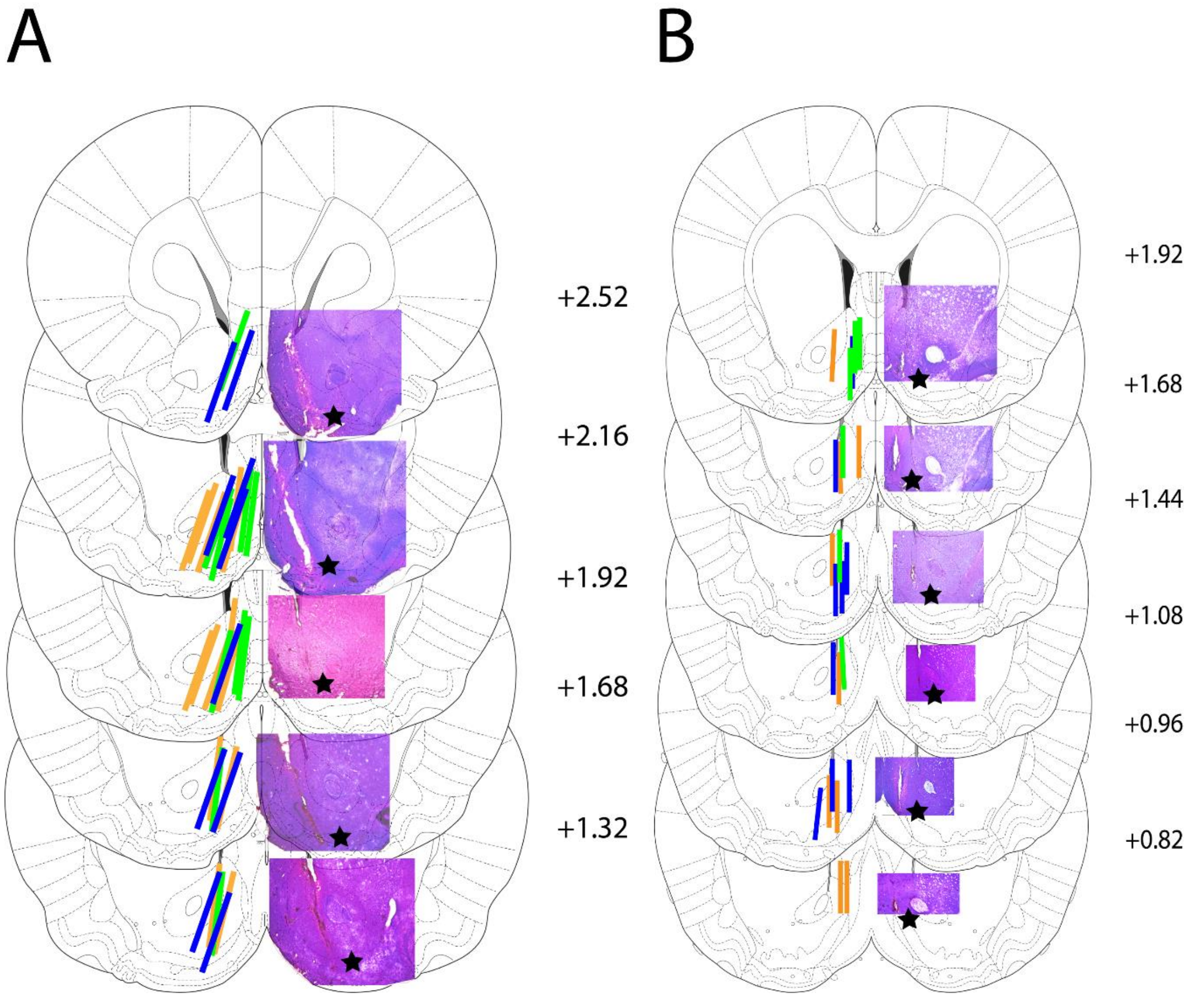

Figure S1. Probes placement. Brain coronal sections showing the placement of the microdialysis probes in Nac of (A) juvenile and (B) adult experimental animals. Line are drawings in the schemas from the atlas of [85]. Vehicle in blue, CP-154,526 in orange and CP-154,526 + Forskolin in green. Numbers to the right indicate mm from bregma. The black stars indicate the placement of the microdialysis probes in the dyed tissue.

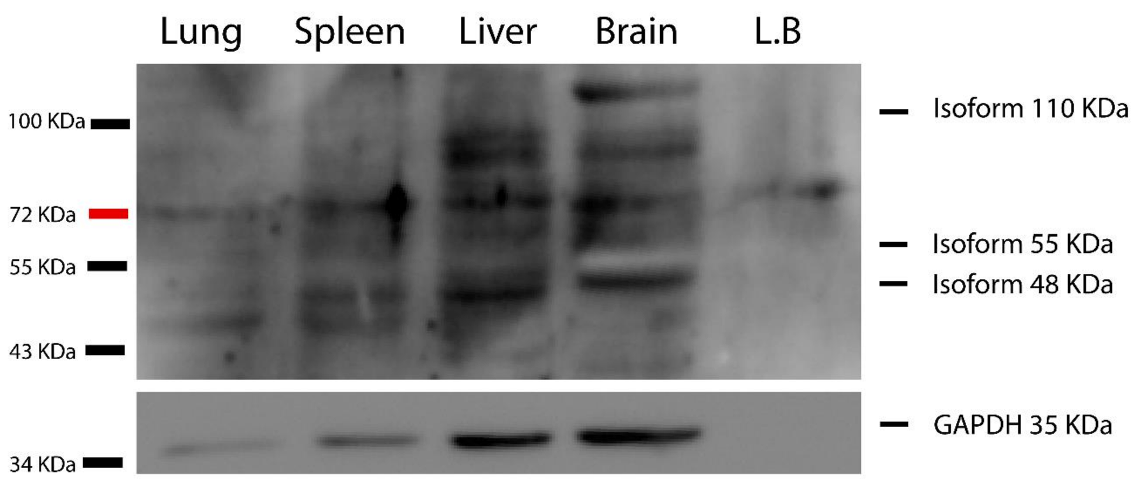

Figure S2. Control of CRF-R1 Western Blot. Comparison of CRF-R1 expression between different tissues using Western Blot. CRF-R1 predicted band size is 48 kDa according to manufacturer instructions. We identified four bands in different tissues but only three isoforms of CRF-R1 were present in the positive control (brain) and absent in the negative control (Lung, spleen, liver and loading buffer). The two bands below of 55 kDa and the band upon 100 KDa. The band above 35 kDa corresponds to GAPDH protein level.

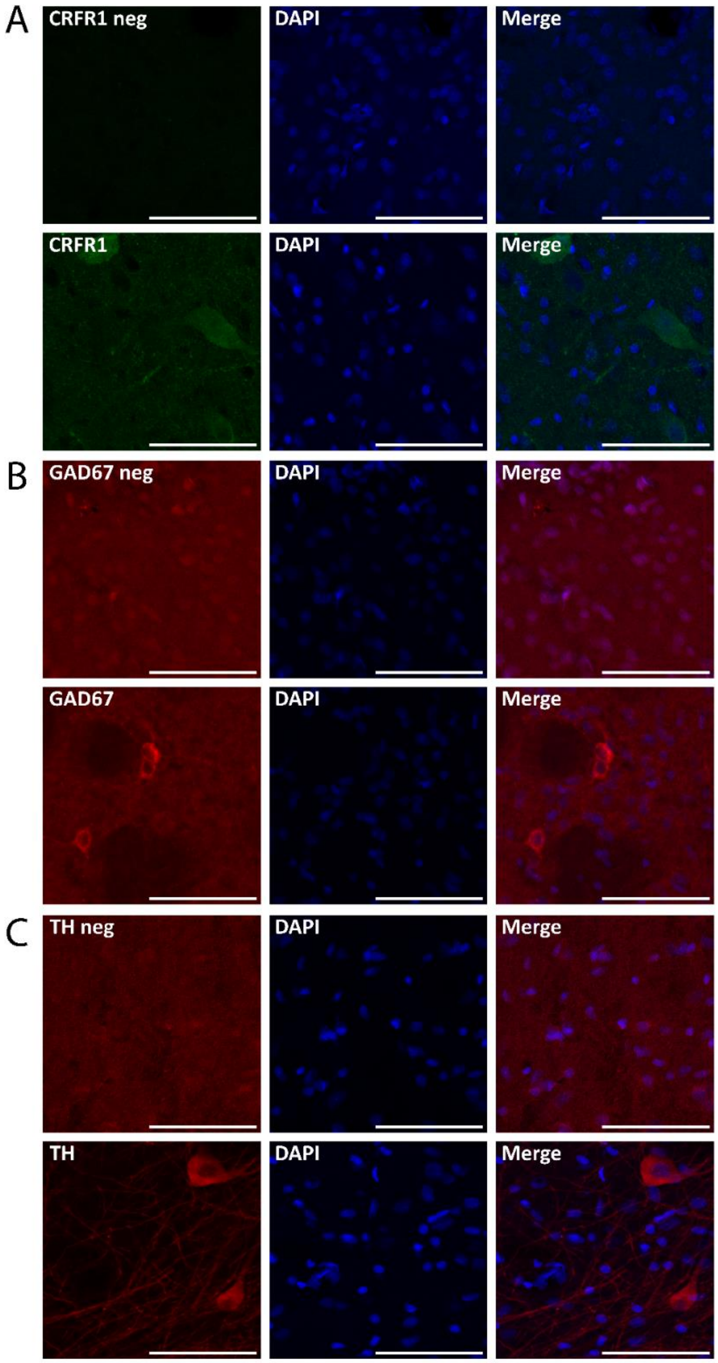

Figure S3. Positive and negative immunofluorescence controls. Figure shows confocal microscopy images validating immunofluorescence utilization of antibodies for (A) CRF-R1, (B) GAD67 and (C) TH. Scale bar represents 70 µm. Images (A) and (B) were made on Nac coronal slices, while (C) was made on VTA horizontal slices. Negative controls lack primary antibodies.

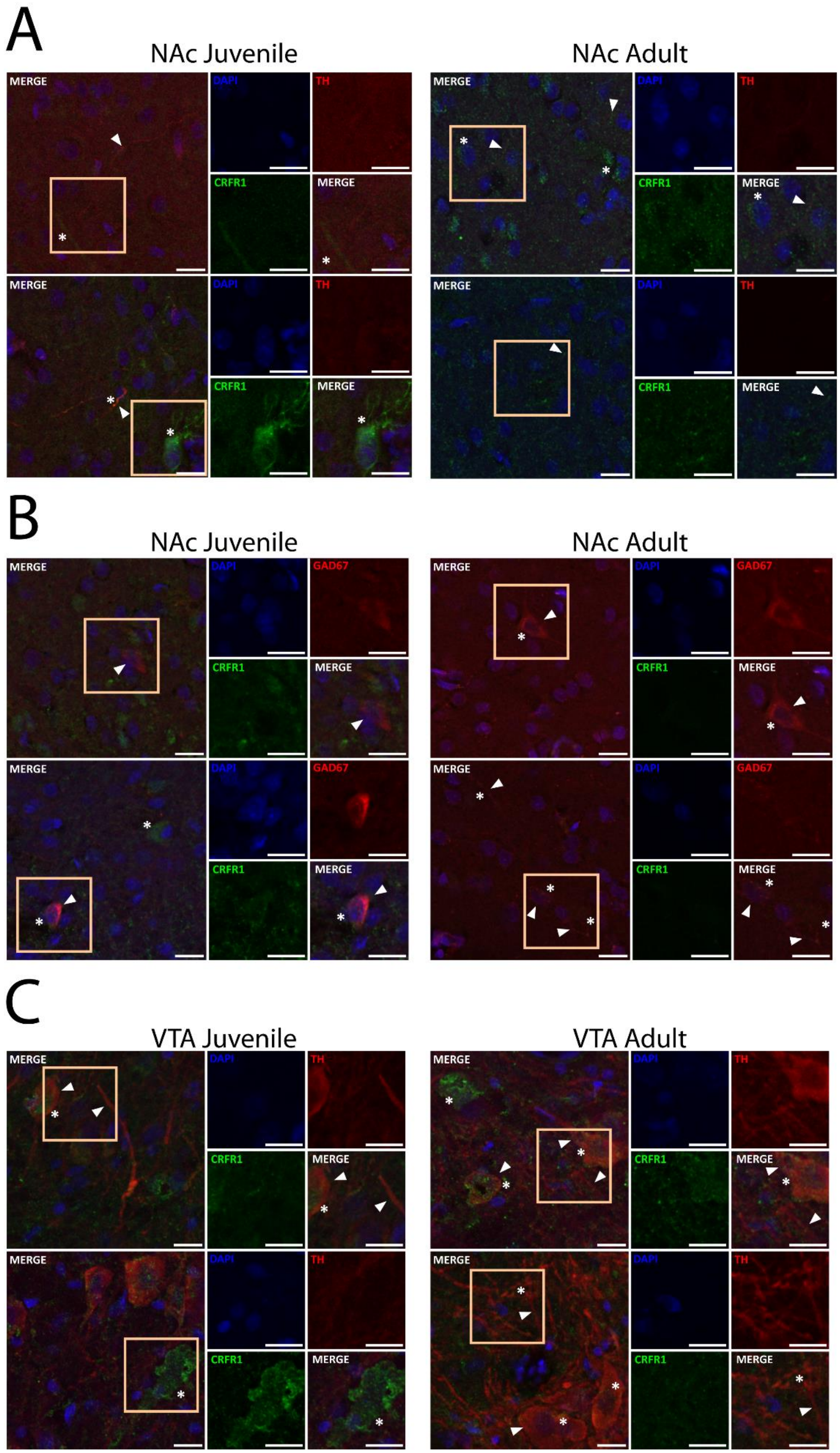
A
NAc Juvenile
NAc Adult
MERGE
DAPI
TH
CRFR1
B
NAc Juvenile
NAc Adult
GAD67
C
VTA Juvenile
VTA Adult

Figure S4. Immunofluorescence showed in Figure 5 raw. Figure shows confocal microscopy raw images before brightness adjustments. The right panel is a 1.3 × zoom of the selected region on the left, showing every separate channel and its respective merge image. The scale bar represents 15 µm in every image. (A) Arrowheads show TH-positive axons, and asterisks show puncta pattern distribution of CRF-R1. Parameters modified for juvenile (upper | lower panels). CRF-R1: SB: 0 | 0, B&C: 1242, 2990 | 791, 4728. For TH: SB: 0 | 0, B&C: 1045, 1571 | 726, 1807 and for DAPI: SB: 0 | 0, B&C: 550, 6100 | 624, 3819. Parameters modified for adult, CRF-R1: SB: 40 | 0, B&C: 1186, 5652 | 1969, 8642. For TH: SB: 40 | 0, B&C: 198, 922 | 991,2714. For DAPI: SB: 40 | 0, B&C: 0, 6635 | 1813, 9624. (B) Arrowheads show GAD67-positive somas and axons, and asterisks show puncta pattern distribu-tion of CRF-R1. Parameters modified for juvenile. CRF-R1: BS: 100 | 0, B&C: -773, 8564 | 879, 4855. For GAD: SB: 100 | 0, B&C: 146, 756 | 286, 1788, and for DAPI: SB: 100 | 0, B&C: 380, 2680 | 753, 6352. Parameters modified for adult, CRF-R1: SB: 100 | 0, B&C: 0, 1532 | 296, 1241. GAD: SB: 100 | 0, B&C: 0, 3410 | 770, 3104. (C) Arrowheads show TH-positive somas and axons, and asterisks show puncta pattern distribution of CRF-R1. Parameters modified for juvenile. CRF-R1: SB: 80 | 0, B&C: 326, 4893 | 575, 5410. For TH: SB: 80 | 0, B&D: -705, 1155 | 289, 936, and for DAPI: SB: 80 | 0, B&D: -1781, 4547 | 461, 1701. Parameters modified for adult. CRF-R1: SB: 80 | 0, B&C: 2030, 11050 | 415, 5578. For TH: SB: 80 | 0, B&C: -210, 1280 | 266, 2065. For DAPI: SB: 80 | 0, B&C: 273, 4896 | 751, 3770. SB, subtract background.

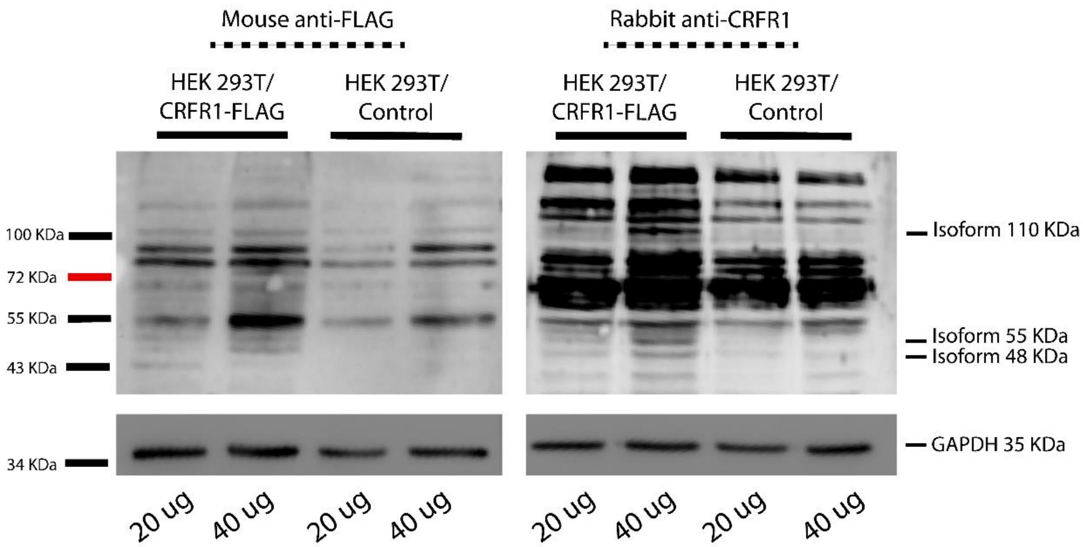

Figure S5. Validation of CRF-R1 antibody in HEK293T. Plates were seeded with HEK293T cells and growth until 80% of confluence. Then, cells were transfected with the plasmid PCDna 3.1 which contains associates the CRF-R1 receptor with a Flag marker using lipofectamine 2000. As a negative control we used HEK 293T non-transfected. 24 hours after transfection, proteins were extracted and quantified to perform a Western Blot as we described in methods section. Membranes were incubated with two primary antibodies, anti-FLAG and anti- CRF-R1. Bands in both membranes were compared. Briefly, we identified nonspecific bands in the control non-transfected cells and in the transfected cells, regardless of the primary antibody used. Nevertheless, there are 2 bands near at 55 KDa in the transfected cells that are not present in the non-transfected ones. In addition, in the Western blot revealed with CRF-R1 there is a band close to 100 KDa that is only in the transfected cells and is not in the control. This experiment shows that regardless the nonspecific bans, there are specific bands for the CRF-R1 that are only recognized by the antibody.

Table S1. List of antibodies used in the immunofluorescences experiments.

**Antibody**	**#RRID or catalog number (cat)**	**Pre-adsorption manufacturer Test**
Rabbit anti CRHR1	AB_2633242	Unspecified
Mouse anti TH	T1299 (cat)	Unspecified
Mouse anti GAD67	MAB5406 (cat)	Unspecified

Goat anti mouse 568	AB_144696	Cross-adsorbed against bovine IgG, goat IgG, rabbit IgG, rat IgG, human IgG, and human serum
Donkey anti rabbit 488	AB_2313584	No antibody was detected against non-immunoglobulin serum proteins. The antibody has been tested by ELISA and/or solid-phase adsorbed to ensure minimal cross-reaction with bovine, chicken, goat, guinea pig, syrian hamster, horse, human, mouse, rat and sheep serum proteins, but it may cross-react with immunoglobulins from other species
